# Terrestrial mountain islands and Pleistocene climate fluctuations as motors for speciation: A case study on the genus *Pseudovelia* (Hemiptera: Veliidae)

**DOI:** 10.1038/srep33625

**Published:** 2016-09-21

**Authors:** Zhen Ye, Pingping Chen, Wenjun Bu

**Affiliations:** 1 Institute of Entomology, College of Life Sciences, Nankai University, 94 Weijin Road, Tianjin, 300071,China; 2 College of Environmental Science and Engineering, Nankai University, 94 Weijin Road, Tianjin, 300071, China; 3 Netherlands Biodiversity Centre – Naturalis, 2300 RA Leiden, The Netherlands

## Abstract

This study investigated the influences of geographic isolation and climate fluctuation on the genetic diversity, speciation, and biogeography of the genus *Pseudovelia* (Hemiptera: Veliidae) in subtropical China and tropic Indo-China Peninsula. Species nucleotide and haplotype diversities decreased with reduction in species distribution limits. The gene tree was congruent with the taxonomy of monophyly, except for four species, *P. contorta*, *P. extensa*, *P. tibialis tibialis*, and *P. vittiformis*. The conflicts between the genes and species tree could be due to long-term isolation and incomplete lineage sorting. Diversification analysis showed that the diversification rate (0.08 sp/My shifted to 0.5 sp/My) changed at 2.1 Ma, which occurred in the early Pleistocene period. Ancestral area reconstruction suggested that subtropical species possibly evolved from the tropics region (i.e., Indo-China Peninsula). Results implied that narrow endemics harbored relatively low genetic diversity because of small effective population and genetic drift. Radiation of subtropical *Pseudovelia* species was rapidly promoted by Pleistocene climate fluctuations and geographic isolation. The acute rising of the Hengduan Mountain with the entire uplift of the Qinghai–Tibet Plateau induced the initial differentiation of *Pseudovelia* species. These results highlighted the importance of geographical isolation and climate changes in promoting speciation in mountain habitat islands.

Understanding speciation is fundamental in ecology and evolutionary biology^1^. Furthermore, diversification patterns and potential driving factors must be elucidated with accurate divergence time estimation to predict species/population and historical demographic changes as well as implement effective conservation in future biodiversity^2^.

Geographic isolation and climate fluctuation are major factors that affects the evolution, speciation, and genetic structuring of extant organisms^3^,^4^. Mountainous subtropical China and adjoining tropical regions possess high degrees of biodiversity and endemism^5^,^6^; these regions include the Hengduan mountain region, which divides the two-tier terrain of China, that is, high elevation in the west and relatively low elevation in the east^7^. In vast areas of subtropical China, numerous scattered mountains (e.g., Nanling, Wuyi, Dabie mountains) resemble natural “terrestrial islands”, which exhibit spatial isolation on restricted land masses and are regarded as ideal natural laboratories for studying speciation of endemic terrestrial plants and animals^8^. Generally, mountains present a wide range of microhabitats with different ecological conditions from the surrounding landscape, which develops to form unique and endemic species with small population size within clearly defined geographical boundaries. Small effective population sizes as well as geographical and ecological isolation in mountains are essential factors that shape the genetic make-up of narrow endemics and thus confer sensitivity to extinction events. The genetic diversity of narrow endemics also determines the future ability of the species to survive^9^. In contrast to that of widely distributed species, speciation of mountain species with restricted distributions and small effective population sizes have been less investigated from a phylogeographic point of view.

Pleistocene glaciations or Ice Ages are recent geo-historical events with major global impact on biodiversity; these events represent the largest expansion of cold climates since the Permian period 250 million years earlier^10^. Glacial influences on the environment vary depending on geographical region. Although most lowland areas of subtropical China and adjoining tropical regions have never been covered by ice sheets, mountainous regions with relatively high altitudes probably experienced strong, cooler, and drier glacial climates as well as major biotic shifts during the Pleistocene^11^. Accumulating evidence suggested that Pleistocene climate oscillations seriously affect the geographic distribution of mountain species and patterns of intraspecific genetic variations^3^,^12^. However, a consensus has not been established regarding the importance of Pleistocene glaciations for inducing mountain speciation in Asia^13^. This phenomenon could be due to the intrinsic properties of existing models. For example, mountain plants (e.g., trees), with relatively long generation times, cannot result in species-level divergence during short duration of climate cycles^14^. For terrestrial mountain animals with relatively short generation times, the strong dispersal ability induces them to solely respond by shifting their ranges toward ecologically suitable areas^15^. Another reason is that the complex climate changes in Asia might have various influences on speciation by comparison with the well-known history of climate glaciations in North America and western Europe^11^.

Mountain stream invertebrates exhibit short generation times and restricted dispersal abilities and are thus suitable models for determining the effects of geographic isolation and Pleistocene climate fluctuations on speciation. This study focuses on the genus *Pseudovelia* (Hemiptera: Veliidae), which is a relatively species-rich genus of mountain stream invertebrates; this genus contains 22 recognized species in subtropical China and tropical Indo-China Peninsula^16^,^17^,^18^,^19^,^20^. *Pseudovelia* species live in quiet and secluded habitat behind boulders of mountain streams at relatively high altitudes^21^. Of the 22 recognized species and two undescribed species, only *P. tibialis tibialis* is widely distributed and most species have adapted to localized distributions (i.e. *P. buccula*, *P. contorta*, *P. feuerborni*, *P. extensa*, *P. intonsa*, *P. pusilla*, *P. sexualis*, *P. sichuanensis*, *P. spiculata*, *P.* sp2, *P. ullrichi*, *P. vittiformis*) or restricted to single mountain massifs (i.e., narrow endemics including *P. anthracina*, *P. fulva*, *P. globosa*, *P. hsiaoi*, *P. longiseta*, *P. longitarsa*, *P. lundbladi*, *P. piliformis*, *P. recava*, *P.* sp1, *P. taiwanensis*)^16^,^19^,^20^. The use of this genus as a study model can clearly define the diagnostic characteristics among closely related species as well as species boundaries; hence, this model can eliminate ambiguous taxonomic problem and enables the establishment of a priori precise designation of species for potential analysis of conflicts between gene and species tree (i.e., incomplete lineage sorting and hybridization)^10^.

In this study, we used a combination of phylogeography, interspecific phylogeny, and ecological niche modeling (ENM) methods as well as multilocus genetic markers to elucidate the effects of geographic isolation and Pleistocene climatic oscillations on the genetic diversity, speciation, and biogeography of *Pseudovelia* in subtropical China and tropical Indo-China Peninsula. In particular, we attempt to test the following hypotheses: (1) narrow endemics are related to relatively low genetic diversities, which are potentially sensitive to extinction events; (2) radiation of subtropical *Pseudovelia* species is boosted in a rapid and recent diversification event, mostly promoted by Pleistocene climate fluctuations and geographic isolation; (3) the phylogeny of recently radiated species may reveal conflicts between the gene and species tree on the account of the evolutionary process (i.e., incomplete lineage sorting), and (4) subtropical *Pseudovelia* species possibly evolve from the tropics region (i.e., Indo-China Peninsula) as influenced by the uplift of the Hengduan mountains.

## Results

### Species genetic diversities in different scales of distribution limits

**Figure 1:**
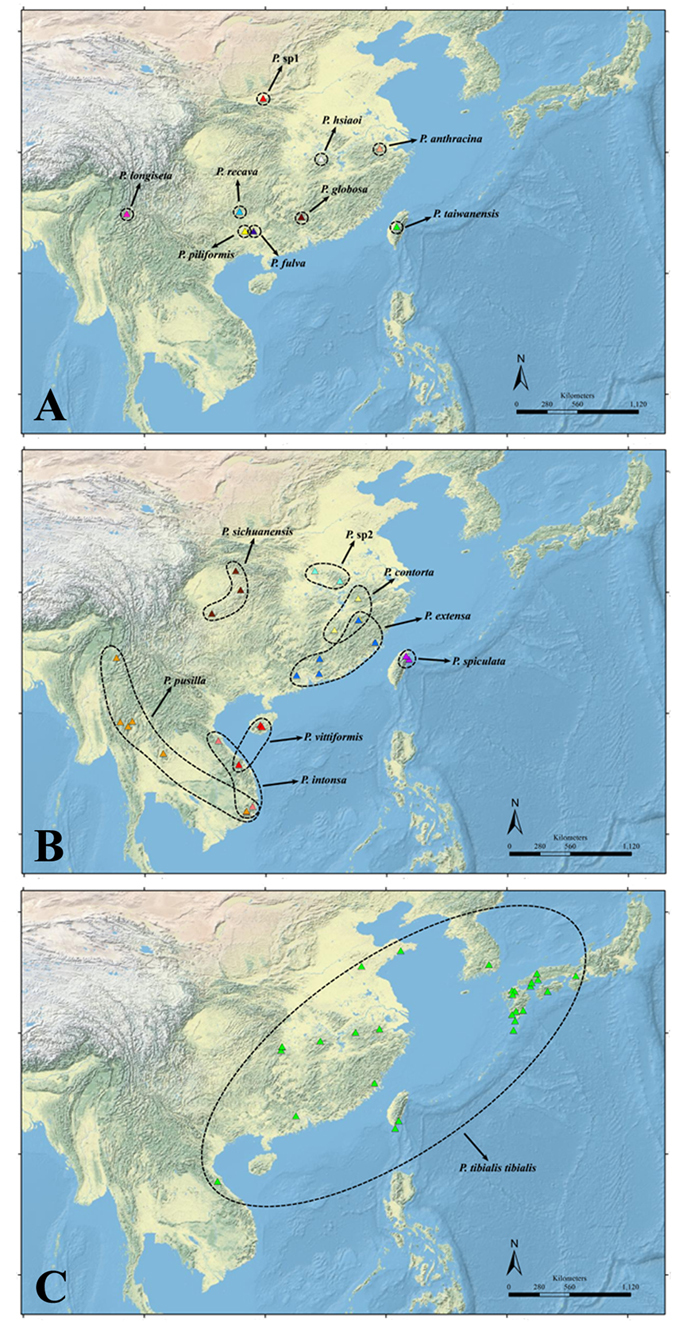
Map of subtropical China and tropic Indo-China Peninsula showing all distribution records of 18 *Pseudovelia* species in this study. (**A**) Narrow endemics (**B**) Localized distribution species (**C**) Wide distribution species. Figure was generated in ArcGIS 10 (Environmental Systems Research Institute, http://www.esri.com/).

Protein-coding regions (1378 bp) were obtained from 285 individuals of *Pseudovelia* species, including sections of the COI (699 bp) and COII (679 bp) genes. A total of 128 unique haplotypes were derived from concatenated COI + COII sequences among all individuals. The 332 polymorphic sites included 18 singleton variables and 314 parsimony informative sites. The nucleotide and haplotype diversities of *Pseudovelia* species ranged from 0.00000 to 0.01892 as well as from 0.000 to 1.000, respectively (Table 1). Both species nucleotide and haplotype diversities decreased with reduction in species distribution limits (Figs 1 and S1), with the lowest value (*Hd* = 0*, π* = 0) found in narrow endemics (Table 1).

### Phylogenetic reconstruction based on mitochondrial and nuclear data

**Figure 2:**
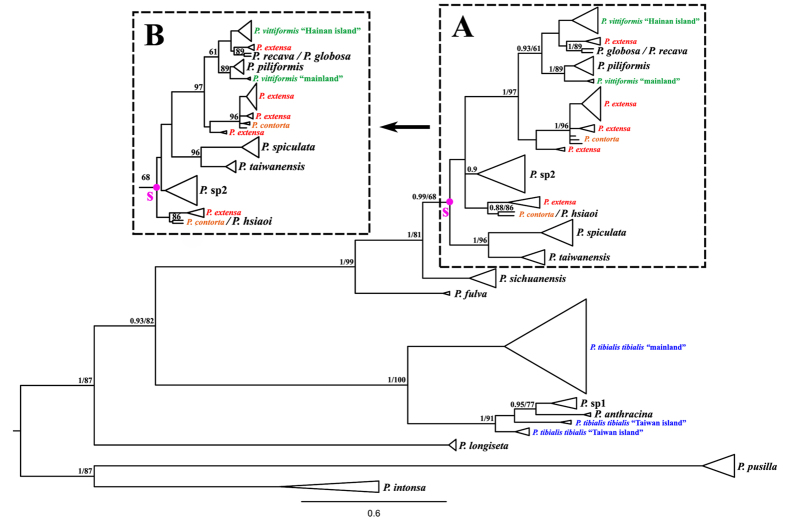
Phylogram of the gene tree based on mitochondrial DNA. The color species name representing non-monophyly of the four species in the gene tree. Purple circles mark nodes S group representing “Node S Species Composition” (NSSC). The phylogenic relationship within the NSSC species showed different topological structure in the two analytical methods: (**A**) Bayesian inference (BI); (**B**) Maximum likelihood (ML). Posterior probabilities/ML bootstrap percentages under 0.8/50 are not showed.

For the mitochondrial data, both Bayesian inference (BI) and maximum likelihood (ML) results revealed compatible tree topologies (Fig. 2), which are largely congruent with previous taxonomic studies based on monophyly^19^,^20^, except for four species, namely, *P. contorta*, *P. extensa*, *P. tibialis tibialis*, and *P. vittiformis*; of these species, *P. extensa* was strongly polyphyletic in the gene trees (Fig. 2). The phylogenic relationship within the “Node S Species Composition” (NSSC) species also showed moderately different topology in the two analytical methods (BI and ML) (Fig. 2A,B). For the nuclear data, the consistently resolved topology (BI/ML) indicated moderate conflict with the mitochondrial gene tree (Fig. S2). All species were strongly monophyletic in the nuclear gene trees (Fig. S2). Of which, two pairs of species (i.e. *P. contorta*/*P. extensa* and *P. anthracina*/*P.* sp1) exhibited the identical nuclear sequences and shared the same haloptype respectively (Fig. S2).

### Species tree analysis and divergence time estimation

**Figure 3:**
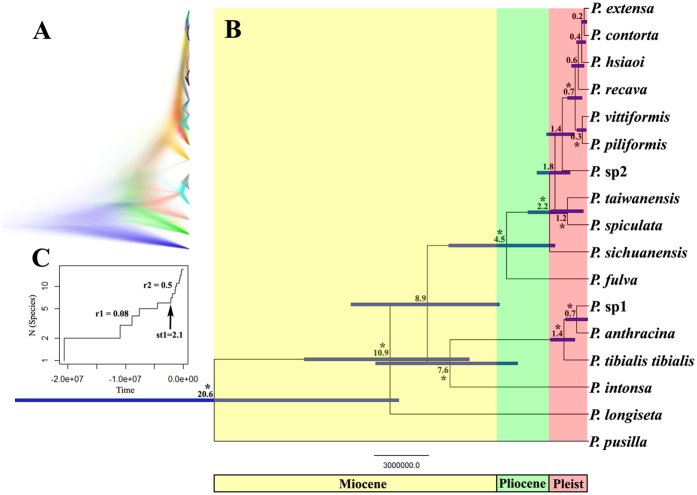
(**A**) Cloudogram of the multilocus species tree analyses performed using the algorithm implemented in *BEAST. (**B**) Chronogram of the same species tree showing estimates of divergence times obtained with BEAST. Blue bars are 95% confidence intervals. Numbers at nodes are the average estimates of divergence times in myr ago. Asterisks indicate nodes with posterior probability >90%. (**C**) Lineage Through Time (LTT) plot for *Pseudovelia* species radiation. Relative time is given on the x-axis, number of species is given on the y-axis.

The *BEAST multilocus species tree is largely congruent to the gene tree (Fig. 3A,B). The main difference of both tress is that, in the gene tree, *P. intonsa* is the sister to *P. pusilla*; conversely, in the *BEAST tree, *P. intonsa* is the sister to the ancestor of the *P. anthracina*, *P*. sp1, and *P. tibialis tibialis*. Almost all nodes of the NSSC species were poorly supported on account of polyphyly of the species *P. contorta*, *P. extensa*, and *P. vittiformis* (Fig. 3B). This issue could neither be solved by repeating runs with higher sample frequencies nor with application of simple substitution models^22^ because of some evolutionary processes (e.g., incomplete lineage sorting). Estimating divergence time between the tropic and subtropical species occurred in the late Miocene (i.e. 7.6–20.6 Ma) (Fig. 3B). Most subtropical species were a recent rapid radiation occurred during the Pleistocene (i.e. 0.2–2.2 Ma), especially for the NSSC species (i.e. 0.2–1.8 Ma) (Fig. 3B).

### Diversification analyses

Monte Carlo constant rates (MCCR) test (critical value = −1.94, *P* = 1) and the gamma statistic (value = 2.88, *P* = 0.9) indicated an increase in the diversification rate over time. Of the six models tested, the variable rate yule2rate model with one shift in diversification was selected as the best fit model for *Pseudovelia*, suggesting that the initial diversification rate of 0.08 species per million years (sp/My) shifted to 0.5 sp/My at 2.1 Ma (Table S1). The Lineage Through Time (LTT) plots also indicated that *Pseudovelia* did not exhibit a constant speciation rate: an acceleration in speciation of the NSSC species lineages occurred in the Pleistocene (Fig. 3C).

### Exploring potential reasons influencing species monophyly

**Figure 4:**
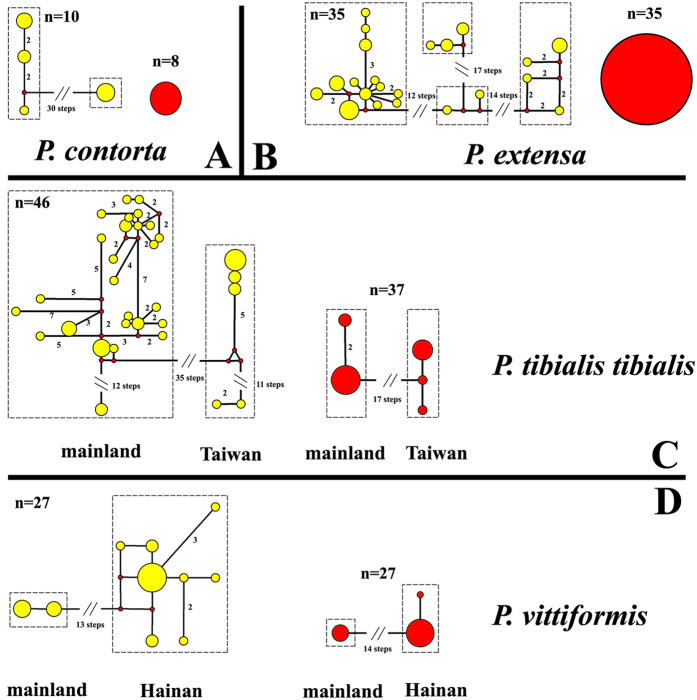
Median joining haplotype network constructed using Network. Haplotype circle size denotes the number (n) of sampled individuals. Yellow/Red colors correspond to mtDNA/nrDNA respectively. Numbers of base pair changes (no number = 1 bp) are given. (**A**) *P. contorta*; (**B**) *P. extensa*; (**C**) *P. tibialis tibialis*; (**D**) *P. vittiformis*.

Our examination displayed no distinctly morphological variations among geographic populations, especially on the important diagnosis (i.e. structure of abdominal segment VIII) (Fig. S3); this finding is consistent with previous taxonomic studies and thus confirms the species status. However, *P. tibialis tibialis* and *P. vittiformis* showed slightly intraspecific morphological variations (i.e. color and body size) between the mainland specimen and their relatives from nearby islands (i.e. Taiwan and Hainan) (Fig. S3). The results of network also showed that both mtDNA and nrDNA haplotypes in *P. tibialis tibialis* and *P. vittiformis* respectively exhibited distinct genetic barrier between mainland and island populations that is separated by Taiwan Strait and Beibu Gulf (Fig. 4C,D). By contrast, the scattered mtDNA haplotypes, non-variation, and identity of nrDNA sequences in *P. contorta* and *P. extensa* suggested that incomplete lineage sorting or hybridization potentially influenced the non-monophyly of the two species (Fig. 4A,B), not geographical isolation. The results of the Joly, McLenachan and Lockhart (JML) run are given in Table S2. All species pairs exhibited genetic distances that are not significantly lower than expected (p > 0.1). Thus, we cannot reject the hypothesis of incomplete lineage sorting in any cases.

### Paleoclimate niche modeling reconstruction

The high Area Under Curve (AUC) value was obtained from the currently potential distribution (AUC = 0.890), indicating good predictive model performance. The current niche predicted the ancestral distribution of the NSSC species, which showed a potentially continuous range located in the Wuyi mountains, Taiwan island, and scattered in coastal areas of southern China (Fig. S4). When projecting the current niche into historical climate conditions, during the LIG period, the suitable climate space continued to stay *in situ*, but a relatively continuous range expand moderately in the coastal areas of southern China (Fig. S4). During the ice age (LGM period), the species’ potential range greatly contracted and habituated in the two segregated refugia (i.e. the Guangxi and the Taiwan space) under the Community Climate System Model (CCSM) model (Fig. S4).

### Ancestral area reconstruction

**Figure 5:**
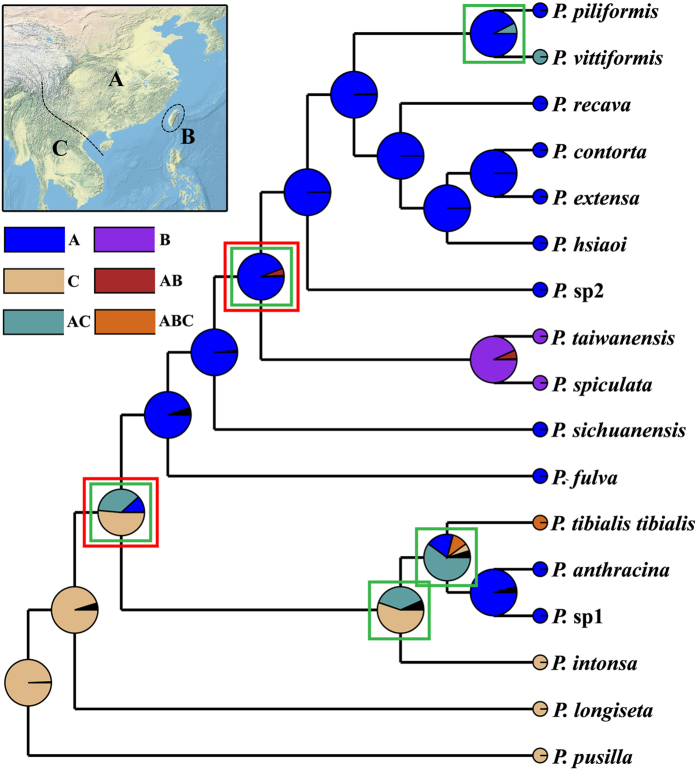
Ancestral area optimizations performed with Bayesian binary Markov chain Monte Carlo (MCMC) (BBM). Pie chart at each node is conducted from BBM implemented in RASP 3.0^64^. Area labels, as stated in the map: (**A**) subtropical China; (**B**) Taiwan island; (**C**) tropic Indo-China Peninsula. Map was modified in ArcGIS 10 (Environmental Systems Research Institute, http://www.esri.com/). The green and red rectangles represent dispersal and vicariance events respectively.

**Figure 6:**
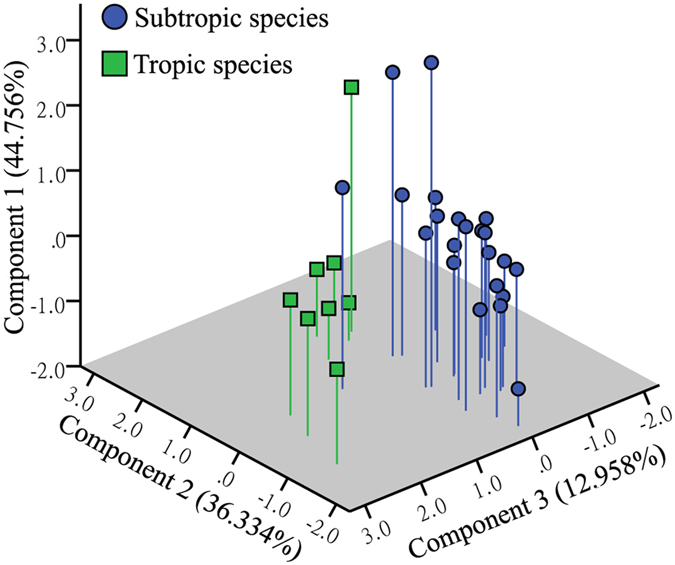
Principle component analysis of seven bioclimatic variables associated with occurrence of subtropical and tropic species.

The two runs of the Bayesian Binary Method (BBM) analysis for the major nodes of the tree produced identical results (Fig. 5), suggesting that *Pseudovelia* species in subtropical China were younger, which probably originated somewhere in tropic Indo-China Peninsula. The Principal Components Analysis (PCA) of pooled environmental variables revealed reduced significant components, defining a realized niche space occupied by subtropical and tropic species (Table 2, Fig. 6). The first two components of the PCA were significant and explained 81.09% of the overall variance. The first component (PC-1) was closely associated with precipitation while the second (PC-2) was associated with temperature (Table 2). The climate space occupied by subtropical species departed from that occupied by tropic species with respect to components, 1 and 2 (Fig. 6). The relatively disjuncted positions in climate space suggested that niche space might diverge between the subtropical and tropic species (Fig. 6).

## Discussion

### Low level of genetic diversities in narrow endemics

Species genetic diversity is an important index for conservation biology^23^. The variation of gene might determine whether population of species could adapt to abiotic and biotic factor changes for survival. Two parameters important to species’ genetic diversity are the influence of effective population size and genetic drift. Effective population size (*Ne*) is usually linked to species distribution patterns and limits, which is a crucial metric because it integrates the genetic effects of life history variation on microevolutionary processes^24^. As *Ne* decreases, genetic drift erodes genetic variation, elevates the probability of fixation of deleterious alleles, and reduces the effectiveness of selection, which reduces overall fitness and limit adaptive responses. These genetic changes can drive a threatened species closer to extirpation^24^. A low level of genetic diversity is commonly expected for narrow endemic species^25^. Our analysis revealed that the widely distributed species, *P. tibialis tibialis*, exhibited the highest genetic diversities (*Hd* = 0.972; *π* = 0.01892) (Table 1). As the species distribution limits reduced from localized scale to single mountain massifs, both species nucleotide and haplotype diversities decreased (Fig. S1). In subtropical China, extremely low diversity (*Hd* *=* 0*, π* *=* 0) had been detected in three of narrow endemics, i.e., *P. globosa*, *P. hsiaoi*, and *P. recava*, which only inhabited in Nanling, Jiugong, and Maolan mountains, respectively (Table 1). This genetic pattern might results from the combined effects of genetic drift and/or isolation^25^. These two factors, together with the relatively small effective population size, potentially eroded genetic variation over time. In fact, geographical isolation could inhibit gene flow and accelerate the effects of random genetic drift, which undoubtedly make narrow endemics more sensitive to extinction events (e.g., human disturbance and natural disaster). Conservation measures therefore should focus on the establishment of nature reserves and create adequate habitat to ensure the viability and long-term sustainability of narrow endemics populations.

### Pleistocene climate oscillations and terrestrial mountain islands inducing a rapid radiation

The low divergences among haplotypes of the NSSC species suggest that these species originated in a recent and rapid radiation (Fig. 2). Our estimated divergence time in the species tree also showed that the NSSC species underwent a recent radiation, which occurred during the Pleistocene (i.e., 0.2–1.8 Ma) (Fig. 3B). The test for heterogeneity of the temporal diversification rate identified the yule2rate model that fit the LTT plot (Fig. 3C). The rate change (0.08 sp/My shifted to 0.5 sp/My) was at 2.1 Ma, which was during the early Pleistocene. Clearly, a high speciation rate has characterized the diversification of NSSC species lineages through time. In general, the average speciation rate in insects was proposed to be 0.16 species per My^26^. This fact suggested that the NSSC species was an unexpected event from what is known about the insect group in continental fauna with such fast diversification rate, because this situation was mostly found in the examples of island species radiation, such as Hawaiian crickets^27^ and Japanese islands ground beetles^28^. This comparison indicated that relatively isolated mountains that resemble natural ‘terrestrial islands’ might occupy identical speciation rate compared with the island fauna radiation.

Many speciation instances had suggested three major promoters of rapid radiation: the appearance of a key innovation that allowed the exploitation of previously unexploited resources or habitats^29^, the availability of new resources^30^, and the availability of new habitats, i.e., a rare colonization event or drastic environmental changes^10^,^14^. In the case of the NSSC species, we found no evident key innovation distinguishing this group from other *Pseudovelia* species. We have no data concerning internal morphology and physiology. Additionally, our observational data showed that the NSSC species had ecological requirements similar to those of other *Pseudovelia* species, which did not indicate the presence of any key innovations. There was also no indication of any new resource that could be specifically exploited by the NSSC species. Based on the ENM reconstruction of ancestral NSSC distribution (Fig. S4), we explored the possibility that drastic environmental changes during the Pleistocene climate oscillations that mediated the radiation of the NSSC species. Based on our results of species divergence time estimation and LTT plots during the Ice Ages, particularly since the late Pleistocene (i.e., 0.9 Ma), very rapid radiation probably occurred in the NSSC species group from 0.7 to 0.2 Ma (Fig. 3B). During this time, subtropical China experienced three glacial periods (i.e., Kunlun, Zhonglianggan and Guxiang glacial periods) and two interglacial periods^31^. The rotation of alternating glacial-interglacial induced large climate oscillations in temperature and rainfall, especially for the mountain regions^31^. Our ENM model simulated the climate oscillations in the late Pleistocene (LIG and LGM) to predict the variation of ancestral suitable habitat of the NSSC species. The results showed that the ancestor contracted mostly into the southern refugia during the cold condition, and expanded at the warm climate condition (Fig. S4). Here, we proposed that during the Pleistocene climate oscillations, the ancestor of the NSSC species might have been forced into ongoing cycles of retreating into, and the re-expansion from, refugia. Under the recurrent, extremely unsuitable climate conditions, the isolation of small populations by mountains over many generations might have promoted speciation and the fixation of morphological traits.

### Underlying factors of discordance between gene and species tree

The conflict between gene and species tree mainly account for the non-monophyly among closed related species. Our gene tree revealed that the four species, *P. contorta*, *P. extensa*, *P. tibialis tibialis*, and *P. vittiformis* were not monophyly in the mitochondrial gene tree (Fig. 2). The taxonomic and morphological studies showed that these species status, based on the important diagnostics of genital morphology, were well supported (Fig. S3). The phylogeographic analysis indicated that the non-monophyly of these four species was potentially caused by two kind effects of evolutionary processes, i.e. geographic isolation and incomplete lineage sorting. For the species *P. tibialis tibialis* and *P. vittiformis*, the mitochondrial and nuclear data both revealed distinctly isolated genetic groups (i.e. island and mainland populations) divided by clearly geographical barriers (i.e. Taiwan strait and Beibu Gulf), which strongly supported the long-time isolated event between the island and mainland populations (Fig. 4C,D). The populations inhabited in the Taiwan and Hainan islands situated 230 and 240 km respectively away from their relatives of mainland. The islands usually shaped local environments, such as the unique microclimate and derived new habitats. As new habitats formed in the Pleistocene, the islands were colonized by *P. tibialis tibialis* and *P. vittiformis* from the mainland. The founders adapted to the new environment, and the long geographical isolation inhibiting gene flow from their mainland ancestral populations had resulted in the high degree of gene differentiation observed today. Under the circumstances, the islands population probably could be easily distinguished by some intraspecific morphological variations from their mainland relative. This situation is also found in these two species, which showed that the island specimens were larger (*P. vittiformis*) and darker (*P. tibialis tibialis*) than their relative from mainland (Fig. S3). Therefore, we proposed that the long-time isolation among population would contribute to the high genetic divergence that would result to non-monophyly in the gene tree. Furthermore, we could imagine that the geographical isolation would lead to the formation of a new taxa if this isolated effect kept enough time.

It was in fact difficult to differentiate between the hybridization and incomplete lineage sorting. Hybridization, the transmission of alleles from one species into the gene pool of second species^32^, usually happened in hybrid zone and is increasingly believed to have played a role in the diversification of animals^33^. Hybridization for the observed mtDNA pattern in closely related species, had been proposed for various animal organisms^34^, which also had been shown to contribute to speciation^35^. However, in the case of *P. contorta* and *P. extensa*, the visible difference in their genital morphology, and the absence of specimens identifiable as hybrid zones, did not support hybridization^36^. Based on the analysis of JML, incomplete lineage sorting was the more likely explanation for the observed non-monophyly of mtDNA patterns in the case of the *P. contorta* and *P. extensa*. Incomplete lineage sorting, or the retention of ancestral polymorphism, was relatively common among recent and rapidly radiating species as these species had not yet have time to fix itself for alternative haplotypes or alleles^37^, which led to the phenomenon that the descendant lineages were expected to share polymorphic alleles with the ancestral population for some time. Once lineage sorting was complete in the closed related species, the phylogenetic relationships of incipient species typically progressed from initial polyphyly through paraphyly and reached monophyly. Thus, the relatively young species might tend to appear polyphyletic or paraphyletic owing to incomplete lineage sorting^38^,^39^. This evolutionary process could affect mitochondrial genes particularly in closely related species where hardly any diversification in nuclear genes was found^40^, which was well fitted to the evidence of the identical nuclear sequences (ITS1 + 5.8S) between the species *P. contorta* and *P. extensa*. In addition, the morphological studies showed that there was also no intraspecific morphological variation among the different geographic populations of these two species. Therefore, incomplete lineage sorting as an explanation for non-monophyly of the species *P. contorta* and *P. extensa* was reasonable.

### Mountains uplift inducing *Pseudovelia* species initial differentiation

Ancestral state reconstruction strongly supported a tropics region (i.e. Indo-China Peninsula) was origin of subtropical species, suggesting that the presence of the subtropical *Pseudovelia* species in the A area was considered to be the result of subsequent vicariance/dispersals from the ancestral distribution (Fig. 5). The tropic and subtropical species lineage was primarily differentiated in the mitochondrial gene tree (Fig. 2). The divergence time estimation revealed that the tropic species ancestor gave rise to the subtropical species lineage through the vicariance/dispersal events during the late Miocene (about 10.9 Ma) (Fig. 3), which was consistent with the beginning time (i.e. 7–10 Ma) of strong uplift of Hengduan mountains in the south region of Qinghai-Tibet Plateau^41^. The acute rising of the Hengduan mountains with the entire uplift of the Qinghai-Tibet Plateau induced the drastic climatic change in the Hengduan mountains region, which leaded to the weather aridification and disappearance of summer monsoon from the Indian Ocean into mainland Asia^41^.

Currently, the Hengduan mountains are characterized by parallel mountain ridges reaching elevations of over 5000 m a.s.l. and elevational differences from valleys to mountain tops that often exceed 2000 m a.s.l.^42^. The BI and ML mitochondrial phylogenetic trees both revealed that substructures of *Pseudovelia* species are probably located in the subtropical China and tropic Indo-China Peninsula, which showed that the Hengduan mountains have acted as an important geographical barrier preventing gene flow at the two sides of the mountainous region, leading to long-term isolation and *in situ* diversification. Furthermore, this extreme topographic complexity could lead to ecological stratification and heterogeneity of environment^43^. Thus, the climatic differences between lineage-specific niches might have placed ecological constraints on these lineages because each would be adapted to the local conditions. The genetic differentiation resulting from the geographical isolation could be further reinforced and accumulated because of ecological divergence over time. Our PCA analysis also indicated that temperature and water, particularly annual mean temperature (BIO1) and annual precipitation (BIO12), were the most important measured variables controlling the geographic distribution of tropic and subtropical groups (Table 2). Therefore, specific climate ecological factors at the two sides of the Hengduan mountains might also contribute to the restriction of gene flow between tropic and subtropical species, and promoted initial differentiation in these two regions.

## Conclusions

Our study investigated influences of terrestrial mountain islands and Pleistocene climate fluctuations on the genetic diversities, speciation, and biogeography of the genus *Pseudovelia* (Hemiptera: Veliidae) in subtropical China and tropic Indo-China Peninsula. Our results provided evidence that the subtropical *Pseudovelia* species likely evolved from the tropics region (i.e. Indo-China Peninsula) and then experienced an extremely rapid and recent radiation, which was probably promoted by the Pleistocene climate fluctuations and geographic isolation. We also proposed a scenario wherein the evolving narrow endemics in the Pleistocene climate oscillations led to the repeated restriction and expansion of the ranges of the ancestral species of the NSSC species, which might have promoted the fixation of ecological adaptations and morphological traits in the small and isolated mountain refugia populations. Taking this scenario into account, the narrow endemics usually harbored relatively low genetic diversities on the account of the intensive effects of small effective population and genetic drift. This successful example highlighted the importance of geographical isolation and climate change in promoting speciation in mountain habitat islands.

## Methods

### Sampling and laboratory procedures

We obtained 285 individual samples from 16 of the 22 recognized species and two undescribed species in subtropical China and tropic Indo-China Peninsula, in which 15 species covering their entire distribution (Table S3, Fig. 1). All samples were preserved in 95% ethanol and stored in a freezer at −20 °C in the College of Life Sciences at Nankai University (Tianjin, China). Genomic DNA was extracted from the entire body, excluding the abdomen and genitalia, by using a General AllGen Kit (Qiagen, Germany). All individuals were sequenced for the mitochondrial markers COI, COII, and the nuclear marker ITS1 + 5.8S. Polymerase chain reactions (PCR) were performed using specific primers following Ye *et al*.^12^. The PCR procedure for COI, COII, and nuclear markers (ITS1 + 5.8S) included an initial denaturation at 94 °C for 2 min, followed by 31–33 cycles of 30 s at 92 °C, 30 s at 48–52 °C, and 1 min at 72 °C, ending with a final extension at 72 °C for 8 min. All fragments were sequenced in both directions using the HiSeq 2000 sequencing system.

### Species genetic diversities in different scales of distribution limits

Genetic diversities were estimated based on the mitochondrial data of each species by using the number of haplotypes (*Nhap*), haplotype diversity (*Hd*), and nucleotide diversity (*π*), which were all calculated in DNASP 4.0^44^. We then used the genetic diversities for comparison between the narrow endemics and localized/wide distribution species.

### Phylogenetic reconstruction based on mitochondrial and nuclear data

The gene tree of haplotypes was reconstructed using Bayesian inference (BI) and maximum likelihood (ML) methods, as implemented in MrBayes 3.2^45^ and raxmlGUI 1.2^46^,^47^ respectively. For the mitochondrial data, the final alignment was 1378 bp in length, and 129 haplotypes were acquired. Models of nucleotide substitution were tested using Modeltest 3.7^48^, and a corrected Akaike information criterion (AICc) was employed to determine the best-fit model. For the BI analysis, under the HKY + I + G model, two simultaneous runs for 5000000 generations with the first 25% was discarded as burn-in. For the nuclear data, the final alignment was 1344 bp in length, and 23 haplotypes were acquired. BI analysis was employed to reconstruct the phylogenetic trees under the GTR + G model. For the ML analysis, we used GTR + I + G model for both mitochondrial and nuclear data and conducted 1000 bootstrap replicates with thorough ML search.

### Multilocus species tree analyses and divergence time estimation

A Bayesian coalescent-based multilocus species tree approach was used to infer phylogenetic relationships among species, which was implemented in BEAST 1.8.2^49^,^50^. *BEAST required a priori designation of species, which we performed based on the taxonomic and morphological studies^19^,^20^. We incorporated 275 COI + COII sequences of mtDNA and 232 ITS1 + 5.8S sequences of nrDNA to estimate a multilocus species tree. This approach had been shown to outperform the traditional concatenation approaches in that incomplete lineage sorting is taken into account, especially in cases where branch lengths are short^51^,^52^. We excluded the *P. globosa* from the analyses because this species lacked data of nuclear loci. We conducted two runs of 10000000 generations (sample freq = 1000 and 25% burnin) and checked for convergence and normal distribution in Tracer v1.6^53^. Estimating divergence time among the *Pseudovelia* species were also assumed to derived from *BEAST analysis. Divergence time was estimated with an uncorrelated lognormal model and a speciation Yule tree prior with the mutation rate 0.4–0.8%/Ma for COI gene^54^ and 0.4–1.0%/Ma for ITS1 gene^55^, with the chains running for 10 million generations with 25% of the initial samples discarded as burn-in.

### Diversification analyses

We used the “chronopl” function of APE (http://ape-package.ird.fr/) to create an ultrametric species tree in R, and constructed semi-logarithmic LTT plots to visualize the temporal variation in the diversification rates^56^. Birth–death likelihood (BDL) models were used to test the significance of heterogeneity or the consistency of the temporal diversification rate^57^. Akaike information criterion (AIC) scores were computed for the constant-rate and the variable-rate models, including the pure birth, birth-death, yule2rate, yule3rate, exponential and logistic variants of the density-dependent speciation rate (DDL), and variable extinction rate and constant speciation rate (EXVAR) models. The model selection was based on the difference in the AIC scores between the best-fitting rate-constant and rate-variable models. The calculations were performed using laser 2.3^57^. In addition, the MCCR test was used by comparing the empirical gamma statistic with the distribution of gamma statistics of 1,000 simulated incomplete phylogenies to test whether the diversification has decelerated through time.

### Exploring potential reasons influencing species monophyly

Based on the phylogenetic results of mitochondrial data, the four species, *P. contorta*, *P. extensa*, *P. tibialis tibialis*, and *P. vittiformis* exhibited non-monophyly in the gene tree, which also conflicted with the result of species tree (see result). Specially, taxonomy errors, geographical isolation, incomplete lineage sorting and hybridization might be the potential reason^10^,^58^. Therefore, we first re-examined morphological characters of all the sample of these four species to confirm species status. We then used Network 4.6.1.3 (Fluxus Technology, Suffolk, UK) to create intraspecific median-joining networks to visualize the evolutionary relationships between haplotypes using both mtDNA and nrDNA^59^, which might reveal the trace of isolated effects on genetic background. To further distinguish incomplete lineage sorting from hybridization, we used a posterior predictive checking method^60^ implemented in JML 1.3.0^61^ to test whether incomplete lineage sorting or hybridization explained discordant relationships between the gene and species tree. We specifically examined conflict phylogeny within node S of gene tree, namely “Node S Species Composition” (NSSC) (Fig. 2). First, we ran another *BEAST analysis of a subset of the multilocus dataset containing only the NSSC species, using the GTR + I + G and GTR + I model for mtDNA and nrDNA, respectively, which sets for a Markov chain Monte Carlo of 11000000 generations (samplefreq = 1,000 and 10% burnin). Second, we used Modeltest 3.7^48^ to estimate the parameters of the substitution model for the mtDNA dataset. Lastly, we conducted a run of the JML software using the same mtDNA dataset, the locus rate of mtDNA as yielded by *BEAST, and the parameters yielded by Modeltest.

### Paleoclimate niche modeling reconstruction

To test whether ancestral NSSC distribution was influenced by Pleistocene climate fluctuation, we incorporated species coordinates of NSSC as the ancestral distribution. A total of 19 occurrence records were obtained for niche modeling. We chose bioclimatic variables representing annual trends and extreme or limiting conditions. Variables that were highly correlated were excluded from our selection, leaving seven variables summarizing temperature and precipitation that were derived from the WorldClim database^62^, i.e. annual mean temperature (BIO1), mean diurnal temperature range (BIO2), maximum temperature of the warmest month (BIO5), minimum temperature of the coldest month (BIO6), annual mean precipitation (BIO12), precipitation of the wettest month (BIO13), and precipitation of the driest month (BIO14). All the variables were at a resolution of 2.5-arc. We used maximum entropy implemented in Maxent 3.3.3 k^63^ to estimate niches in environmental dimensions. Analysis was ran using default program conditions [cumulative output, convergence threshold (10^−5^)], maximum number of iterations (500). Area Under Curve (AUC) of the Receiver Operating Characteristic (ROC) plot was used for model evaluation. For hindcasting the effect of Pleistocene climatic fluctuations, the current native niche models were calibrated using the above environmental variables and then transferred onto the reconstructed climatic conditions during the LGM (CCSM model) and LIG periods.

### Ancestral area reconstruction

Bayesian binary Markov chain Monte Carlo (MCMC) (BBM) implemented in RASP 3.0^64^ was employed to reconstruct the possible ancestral distribution areas (subtropicals or tropics) of *Pseudovelia*. We used the multilocus species tree inferred by *BEAST as input in the program. The study area was divided into three regions, i.e. subtropical China, subtropical Taiwan island, and tropic Indo-China Peninsula, which are separated by the Hengduan mountain and Taiwan Strait. Ten MCMC chains were ran in two independent analyses for 50000 generations under the F81 + G model. The state was sampled every 100 generations. We then compared climate space occupied by subtropical and tropic species using direct climate comparisons and principal component analysis (PCA), as these methods allowed for quick assessments of the relative positions of species in climate space^65^. We extracted the climate value of for each occurrence of these two species using ArcGIS (ESRI, Redlands, CA, USA). The seven variables occupied by subtropical and tropic species were compared visually in boxplots, and statistically tested using independent sample test in SPSS.

## Additional Information

**How to cite this article**: Ye, Z. *et al*. Terrestrial mountain islands and Pleistocene climate fluctuations as motors for speciation: A case study on the genus *Pseudovelia* (Hemiptera: Veliidae). *Sci. Rep.* **6**, 33625; doi: 10.1038/srep33625 (2016).

## Supplementary Material

Supplementary Information

## Figures and Tables

**Table 1:** Nucleotide polymorphism in each species.

Species	Sample size	Nhap	*Hd*	*π*
Narrow endemics				
* P. anthracina*	4	2	0.667	0.00048
* P. fulva*	5	2	0.400	0.00029
* P. globosa*	10	1	0.000	0.00000
* P. hsiaoi*	5	1	0.000	0.00000
* P. longiseta*	20	4	0.437	0.00035
* P. piliformis*	20	6	0.574	0.00134
* P. recava*	6	1	0.000	0.00000
* P. taiwanensis*	18	5	0.484	0.00064
* P.* sp1	9	4	0.694	0.00323
Localized distribution species				
* P. contorta*	10	4	0.778	0.01285
* P. extensa*	35	22	0.965	0.01189
* P. intonsa*	4	4	1.000	0.01367
* P. pusilla*	10	7	0.867	0.00184
* P. sichuanensis*	20	5	0.526	0.00163
* P. spiculata*	16	8	0.900	0.00152
* P. vittiformis*	27	10	0.812	0.00527
* P.* sp2	20	11	0.905	0.00722
Wide distribution species				
* P. tibialis tibialis*	46	31	0.972	0.01892

*S*, number of segregating sites; NHap, number of haplotypes; *Hd*, haplotype diversity; *π*, nucleotide diversity.

**Table 2:** Principal components analysis (PCA) of bioclimatic variables associated with occurrence of subtropical and tropic species.

Variables	Description	Factor loadings		
		PC-1	PC-2	PC-3
**BIO1**	Annual mean temperature	0.025	**0.994**	−0.022
**BIO2**	Mean diurnal temperature range	−0.657	0.375	0.572
**BIO5**	Maximum temperature of warmest month	−0.305	0.671	−0.641
**BIO6**	Minimum temperature of coldest month	0.240	**0.931**	0.130
**BIO12**	Annual precipitation	**0.973**	0.109	0.151
**BIO13**	Precipitation of wettest month	**0.898**	0.252	−0.254
**BIO14**	Precipitation of driest month	**0.893**	−0.145	−0.253
**Eigenvalue**		3.133	2.543	0.907
**Percentage variance**		44.756	36.334	12.958
**Cumulative percentage variance**		44.756	81.089	94.048

Eigenvalues for the most important variables (>0.8) in PCA are in bold.
