# Supplementary material for: Terrestrial mountain islands and Pleistocene climate fluctuations as motors for speciation: A case study on the genus Pseudovelia (Hemiptera: Veliidae)
Source: Sci Rep. 2016 Sep 21;6:33625. doi: 10.1038/srep33625 (PMC5030487; doi:10.1038/srep33625)

**Terrestrial mountain islands and Pleistocene climate fluctuations as motors in speciation: case study on mountain stream genus *Pseudovelia* (Hemiptera: Veliidae)**

Zhen Ye^1, 2^, Pingping Chen^3^, Wenjun Bu^1**^

1 *Institute of Entomology, College of Life Sciences, Nankai University, 94 Weijin Road, Tianjin, 300071,China.*

2 *College of Environmental Science and Engineering,* *Nankai University, 94 Weijin Road, Tianjin, 300071, China*.

3 *Netherlands Biodiversity Centre – Naturalis, 2300 RA Leiden, The Netherlands.*

** Correspondence: Wenjun Bu, Fax: +86-22-23408957, Institute of Entomology, College of Life Sciences, Nankai University; 94 Weijin Road, Tianjin, 300071, China. E-mail: wenjunbu@nankai.edu.cn

**Table S1 Testing models of diversification in *Pseudovelia* in Greater China and tropic Indo-China Peninsula.**

| Clade | Pure birth  AIC | Birth-death  AIC | Yule2rate  AIC | Yule3rate  AIC | DDL  AIC | EXVAR  AIC | γ Statistc | MCCR test  Critical γ |
| --- | --- | --- | --- | --- | --- | --- | --- | --- |
| *Pseudovelia*  Overall | 436.8453 | 437.3655 | 431.4230* | 434.0005 | 653.2756 | 168400.9 | 2.88  (*P* = 0.99801) | –1.94  (*P* = 1) |

*Best fit model for *Pseudovelia* overall, the first diversification rate of 0.08 shifts to 0.5 sp/My at 2.1 Ma.

**Table S2** List of the observed distances among couples of NSSC species with p-values > 0.1, as inferred by posterior predictive distributions from JML.

| Comparison | minDist | Probability |
| --- | --- | --- |
| *P. extensa* **/** *P. contorta* | 0.00217707 | 0.118757 |
| *P. hsiaoi* **/** *P. contorta* | 0.00435414 | 0.381798 |
| *P. hsiaoi* **/** *P. extensa* | 0.00653120 | 0.600444 |
| *P. piliformis* **/** *P. contorta* | 0.01306240 | 0.663707 |
| *P. piliformis* **/** *P. extensa* | 0.00943396 | 0.480577 |
| *P. piliformis* **/** *P. hsiaoi* | 0.02104500 | 0.932297 |
| *P. recava* **/** *P. contorta* | 0.01161100 | 0.577137 |
| *P. recava* **/** *P. extensa* | 0.00725689 | 0.299667 |
| *P. recava* **/** *P. hsiaoi* | 0.02249640 | 0.944506 |
| *P. recava* **/** *P. piliformis* | 0.00870827 | 0.435072 |
| *P. spiculata* **/** *P. contorta* | 0.02104500 | 0.301887 |
| *P. spiculata* **/** *P. extensa* | 0.01741650 | 0.155383 |
| *P. spiculata* **/** *P. hsiaoi* | 0.01959360 | 0.201998 |
| *P. spiculata* **/** *P. piliformis* | 0.02322210 | 0.413984 |
| *P. spiculata* **/** *P. recava* | 0.02322210 | 0.388457 |
| *P. taiwanensis* **/** *P. contorta* | 0.01741650 | 0.130966 |
| *P. taiwanensis* **/** *P. extensa* | 0.01814220 | 0.189789 |
| *P. taiwanensis* **/** *P. hsiaoi* | 0.01814220 | 0.162042 |
| *P. taiwanensis* **/** *P. piliformis* | 0.02104500 | 0.287458 |
| *P. taiwanensis* **/** *P. recava* | 0.0232221 | 0.376249 |
| *P. taiwanensis* **/** *P. spiculata* | 0.0137881 | 0.419534 |
| *P. vittiformis* **/** *P. contorta* | 0.00943396 | 0.406215 |
| *P. vittiformis* **/** *P. extensa* | 0.0065312 | 0.223085 |
| *P. vittiformis* **/** *P. hsiaoi* | 0.0181422 | 0.876804 |
| *P. vittiformis* **/** *P. piliformis* | 0.00435414 | 0.374029 |
| *P. vittiformis* **/** *P. recava* | 0.0065312 | 0.228635 |
| *P. vittiformis* **/** *P. spiculata* | 0.0203193 | 0.27303 |
| *P. vittiformis* **/** *P. taiwanensis* | 0.0203193 | 0.250832 |
| *P.* sp2**/** *P. contorta* | 0.011611 | 0.189789 |
| *P.* sp2 **/** *P. extensa* | 0.0108853 | 0.208657 |
| *P.* sp2 **/** *P. hsiaoi* | 0.011611 | 0.172031 |
| *P.* sp2 **/** *P. piliformis* | 0.0166909 | 0.54495 |
| *P.* sp2 **/** *P. recava* | 0.0181422 | 0.625971 |
| *P.* sp2 **/** *P. spiculata* | 0.0188679 | 0.209767 |
| *P.* sp2 **/** *P. taiwanensis* | 0.0166909 | 0.138735 |
| *P.* sp2 **/** *P. vittiformis* | 0.0159652 | 0.520533 |

**Table S3** Sample information in each geographic population of *Pseudovelia* species. Asterisks indicate sample species covering their entire distribution.

| **Population** | **Lat.** | **Long.** | **Sample size** |
| --- | --- | --- | --- |
|  |  |  |  |
| ***P. anthracina**** ZJLA | 119.477 | 30.369 | 4 |
| ***P. fulva**** GXWMX | 108.334 | 23.527 | 5 |
| ***P. globosa**** GDNL | 112.994 | 24.692 | 10 |
| ***P. hsiaoi**** HBXN | 114.629 | 29.504 | 5 |
| ***P. longiseta**** YNTC | 98.548 | 24.951 | 20 |
| ***P. piliformis**** GXWMD | 108.337 | 23.522 | 20 |
| ***P. recava**** GZLB | 107.882 | 25.147 | 6 |
| ***P. taiwanensis**** TWNT | 120.888 | 23.893 | 18 |
| ***P.* sp1*** SXHX | 109.835 | 34.494 | 9 |
| ***P. contorta**** JXML | 117.649 | 29.537 | 5 |
| JXYF | 115.680 | 26.924 | 4 |
| JXTM | 117.742 | 27.847 | 1 |
| ***P. extensa**** FJWY | 117.683 | 27.748 | 8 |
| FJYT | 119.059 | 25.889 | 4 |
| GDBL | 114.464 | 24.544 | 10 |
| GDDH | 112.555 | 23.159 | 5 |
| JXLN | 114.426 | 23.308 | 8 |
| ***P. intonsa*** VINT | 106.076 | 17.765 | 4 |
| ***P. pusilla*** YNYJ | 97.656 | 24.612 | 10 |
| ***P. sichuanensis**** CQZX | 107.917 | 30.231 | 10 |
| SCPC | 107.526 | 31.807 | 6 |
| SCXY | 105.539 | 28.271 | 4 |
| ***P. spiculata**** TWFS | 121.613 | 24.752 | 6 |
| TWSA | 121.843 | 24.489 | 10 |
| ***P. vittiformis**** HNWZ | 109.678 | 18.904 | 10 |
| HNYG | 109.563 | 19.038 | 10 |
| VIEA | 107.776 | 15.774 | 7 |
| ***P.* sp2*** AHYX | 116.131 | 30.978 | 10 |
| HNXY | 114.074 | 31.802 | 10 |
| ***P. tibialis tibialis*** AHCZ | 119.474 | 30.369 | 2 |
| FJYT | 119.059 | 25.889 | 2 |
| GDDH | 112.553 | 23.175 | 5 |
| HBTS | 111.404 | 28.918 | 2 |
| HNCD | 111.420 | 28.905 | 1 |
| SDHY | 121.227 | 36.846 | 10 |
| SDMY | 117.972 | 35.557 | 10 |
| TWLJ | 121.056 | 22.781 | 10 |
| TWPD | 120.774 | 22.149 | 2 |
| VTIB | 106.076 | 17.765 | 2 |

**Figure S1** Showing the species genetic diversities in different scale of distribution limits. (A) haplotype diversity (B) nucleotide diversity. Figure was generated in Microsoft Office Excel 2003.


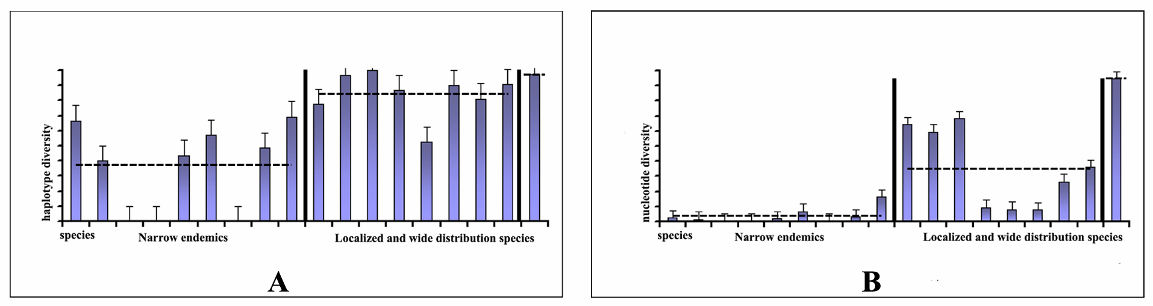


**Figure S2** Phylogram of the gene tree obtained from Bayesian inference (BI) and maximum likelihood (ML) analyses of the nuclear DNA. The number in brackets after species name indicates the number of haplotypes. Numbers of each branch are support values the BI and ML analyses in the order of PP_BI_/BS_ML_.

**
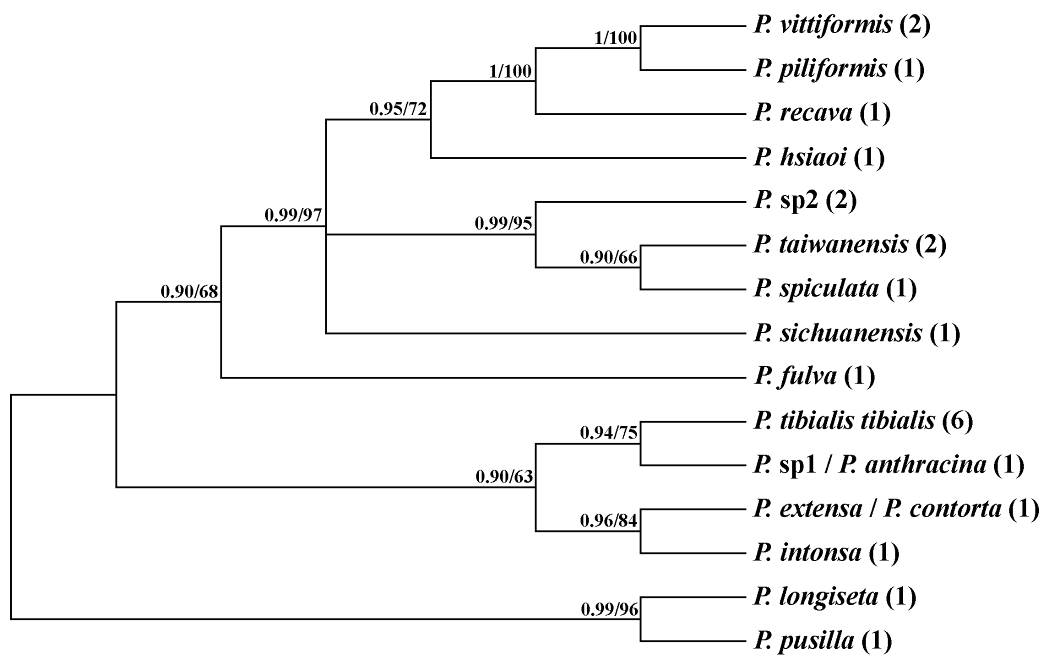
**

**Figure S3** Showing the species diagnostic character and intraspecific morphological variations (i.e. colour and body size) between the mainland and islands specimen. (a) *P. tibialis tibialis* from mainland, scale 1.0 mm; (b) *P. tibialis tibialis* from Taiwan island, scale 1.0 mm; (c) Diagnostic character of abdominal segment VIII in *P. tibialis tibialis*, scale 0.2 mm; (d) *P. vittiformis* from mainland, scale 1.0 mm; (e) *P. vittiformis* from Hainan island, scale 1.0 mm; (d) Diagnostic character of abdominal segment VIII in *P. vittiformis*, scale 0.2 mm. Photographs were taken using a Canon EOS 600D digital camera and modified in Photoshop 7.0 (https://www.adobe.com/).


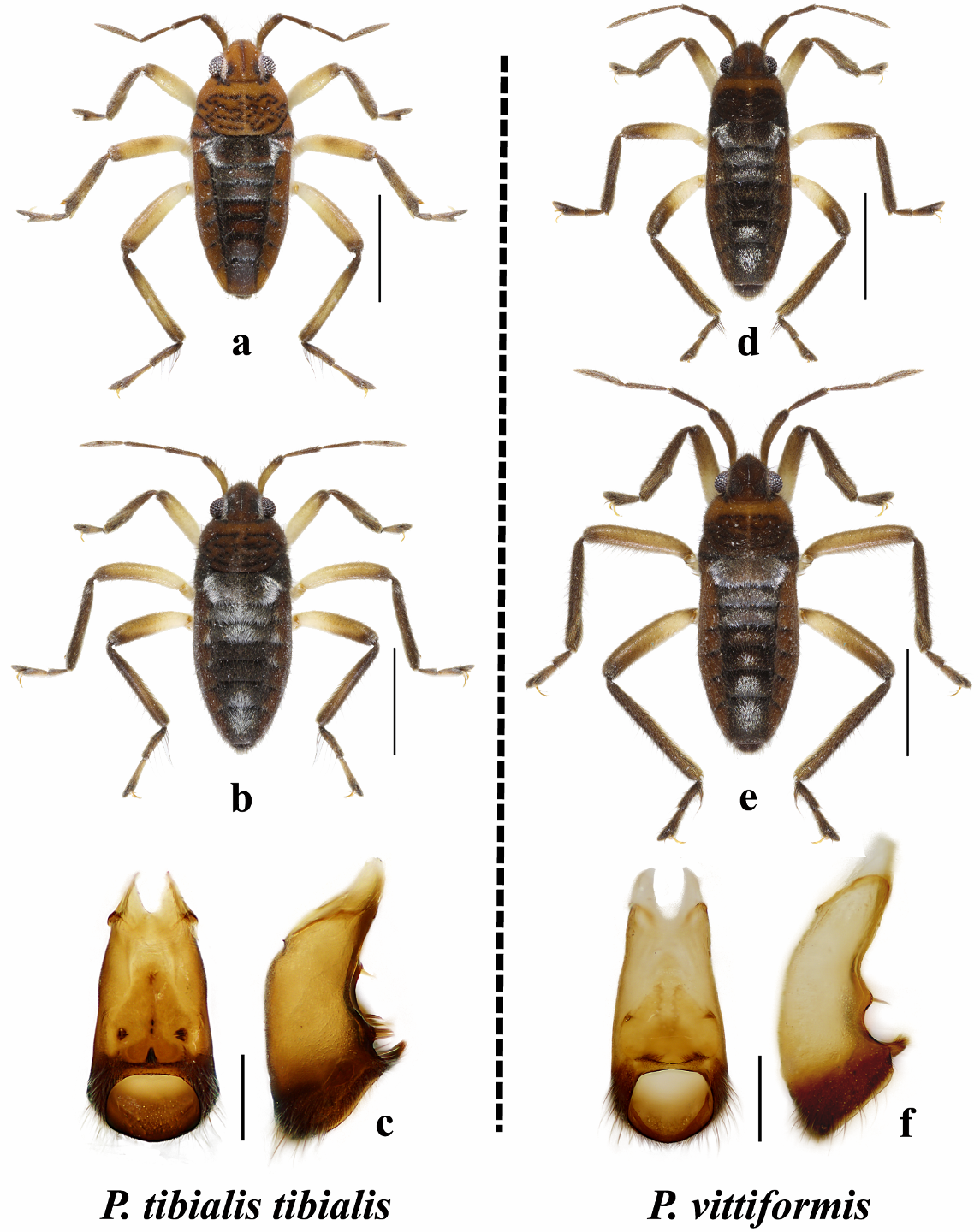


**Figure S4** Hindcasting the current niche model (left panel) onto the LIG (middle panel) and LGM (right panel) periods of ancestor of the NSSC species group in subtropic China using Maxent 3.3.3k^63^. Niche model results were modified in ArcGIS 10 (Environmental Systems Research Institute, http://www.esri.com/).


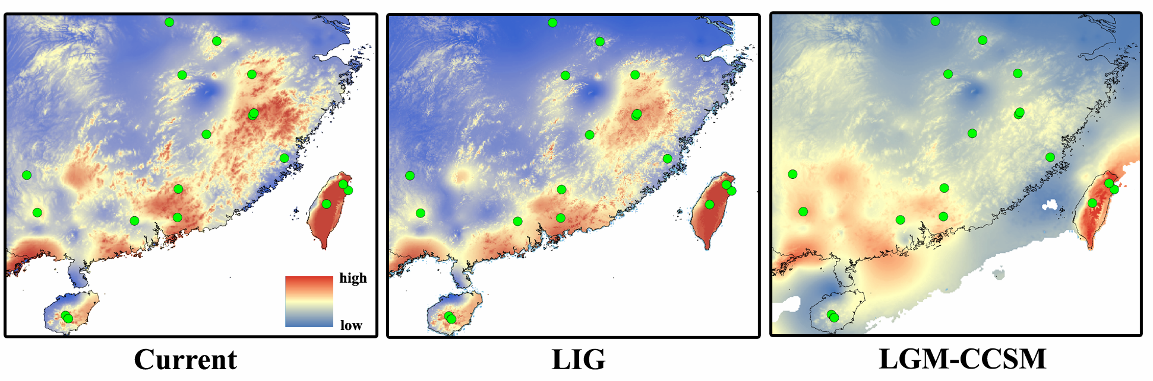

Supplement: Supplementary Information [file srep33625-s1.doc]
